# Bone Mesenchymal Stem Cell-Derived Extracellular Vesicles Promote Recovery Following Spinal Cord Injury via Improvement of the Integrity of the Blood-Spinal Cord Barrier

**DOI:** 10.3389/fnins.2019.00209

**Published:** 2019-03-12

**Authors:** Yanhui Lu, Yan Zhou, Ruiyi Zhang, Lulu Wen, Kaimin Wu, Yanfei Li, Yaobing Yao, Ranran Duan, Yanjie Jia

**Affiliations:** ^1^Department of Neurology, The First Affiliated Hospital of Zhengzhou University, Zhengzhou, China; ^2^Academy of Medical Sciences, Zhengzhou University, Zhengzhou, China; ^3^Department of Radiology, The First Affiliated Hospital of Zhengzhou University, Zhengzhou, China

**Keywords:** extracellular vesicles, bone mesenchymal stromal cells, spinal cord injury, blood spinal cord barrier, pericytes, migration, NF-κB

## Abstract

Mesenchymal stem cell (MSC) transplantation has been shown to represent a potential treatment for traumatic spinal cord injury (SCI). However, there are several obstacles that need to be overcome before MSCs can be considered for clinical application, such as failure of MSCs to reach the spinal cord lesion core and possible tumor formation. Recent studies have suggested that MSC treatment is beneficial owing to paracrine-secreted factors. Extracellular vesicles are considered to be some of the most valuable paracrine molecules. However, the therapeutic mechanism of extracellular vesicles on spinal cord injury has not been studied clearly. Therefore, our study investigated the effect of systemic administration of extracellular vesicles on the loss of motor function after SCI and examined the potential mechanisms underlying their effects. Disruption of the blood-spinal cord barrier (BSCB) is a crucial factor that can be detrimental to motor function recovery. Pericytes are an important component of the neurovascular unit, and play a pivotal role in maintaining the structural integrity of the BSCB. Our study demonstrated that administration of bone mesenchymal stem cell-derived extracellular vesicles (BMSC-EV) reduced brain cell death, enhanced neuronal survival and regeneration, and improved motor function compared with the administration of BMSC-EV free culture media (EV-free CM). Besides, the BSCB was attenuated and pericyte coverage was significantly decreased *in vivo*. Furthermore, we found that exosomes reduced pericyte migration via downregulation of NF-κB p65 signaling, with a consequent decrease in the permeability of the BSCB. In summary, we identified that extracellular vesicles treatment suppressed the migration of pericytes and further improved the integrity of the BSCB via NF-κB p65 signaling in pericytes. Our data suggest that extracellular vesicles may serve as a promising treatment strategy for SCI.

## Introduction

Traumatic spinal cord injury (SCI) causes permanent motor/sensory dysfunction, and even paralysis and death, resulting in a considerable reduction in the quality of life of the patient (Eckert and Martin, 2017). Many studies have shown that the failure of regeneration following SCI relates to molecular and cellular factors, such as the absence of phosphatase and tensin homolog (PTEN) and mammalian target of rapamycin (mTOR), and the presence of Nogo associated with myelin debris, debris-clearing inflammatory cells, and scar-forming cells (Schwab, 2004; Sun et al., 2011). Although considerable research has been carried out on the mechanisms involved, there are still no effective treatments for SCI.

For many years, MSCs have been exploited as an experimental therapy for SCI because of their multidirectional differentiation and potential neuroprotective properties (Xu and Yang, 2018). Although research has shown that MSCs extenuate lesions and improve axon regeneration, several obstacles need to be overcome. For example, it is reported that the majority of MSCs fail to reach the lesion and survive, but accumulate in lung tissue. Thus, the effectiveness of this treatment strategy is limited unless MSCs are directly transplanted into the spinal cord lesion. Fortunately, recent research has suggested that MSCs may play a therapeutic role via the release of paracrine factors and stimulation of host cells, instead of through their differentiation (Caplan and Dennis, 2006; Caplan and Correa, 2011). EV, which are composed of lipid bilayer, are regarded as an important component of MSC-induced paracrine factor secretion. Depending on the size of diameter, EV was divided into exosomes, microvesicles and apoptotic bodies, of which the diameter of exosomes is the smallest, 30–150 nm (Tkach and Thery, 2016; Juan and Furthauer, 2018). EV have the capacity to carry intracellular sorted cargoes, including various proteins, lipids, mRNA, and microRNA, and further target select cells in various ways including delivering functional substances and so on (Camussi et al., 2010; Phinney and Pittenger, 2017).

Several studies have demonstrated that EV participate in therapeutic effects, such as wound regeneration and reduction of neuronal cell death following cerebral ischemia (Goodarzi et al., 2018; Zagrean et al., 2018). However, it is unclear how EV promote motor recovery post-SCI. Therefore, to get a better understanding of the potential mechanisms involved in EV therapy, we further studied how EV protect the BSCB following SCI.

## Materials and Methods

### Isolation and Identification of Extracellular Vesicles

Total bone marrow cells were flushed from the tibias of rats (3-months-old) with Dulbecco’s modified Eagle medium (DMEM; GIBCO). Complete DMEM containing 15% fetal bovine serum (FBS), 100 U/ml penicillin, and 100 U/ml streptomycin was used to culture the bone mesenchymal stem cells (BMSCs). Extracellular vesicles were harvested from passage (P) 3 to P5 of the BMSCs as previously described (Wang et al., 2018). Briefly, exosome-depleted FBS was made by ultra-centrifuging FBS at 120,000 × *g* for 18 h. The culture medium was replaced with an exosome-depleted medium 48 h prior to the isolation of exosomes. The culture supernatants were collected and differently centrifuged at 300 × *g* for 10 min, 2000 × *g* for 10 min, and then 10,000 × *g* for 70 min to remove cells and debris. This was followed by ultracentrifugation at 100,000 × *g* for 70 min to obtain exosomes as pellets. The collected exosomes were resuspended with phosphate buffered saline (PBS) and then ultracentrifuged (110,000 × *g* for 70 min) again to refine the purity of the extracellular vesicles (BMSC-derived extracellular vesicles; BMSC-EV). The remaining medium was extracellular vesicles-free culture medium (EV-free CM). The morphology of the acquired EV was observed by transmission electron microscopy (TEM; Tecnai 12, Philips, Netherlands). Western blot was performed to verify the specific exosome surface markers, including CD9, CD63, and CD81.

### BMSC-EV Labeling

PKH26 dye (Sigma-Aldrich, St. Louis, MO, United States) with a final concentration of 2 × 10^6^ M was incubated with BMSC- EV at 25°C for 20 min followed by an equal amount of 5% bovine serum albumin (BSA; Sigma-Aldrich) to stop the staining reaction. Then, the mixture was resuspended in PBS and the EV were extracted again by ultracentrifugation as described above.

### Animals

All procedures and operations complied with the National Guidelines for the Care and Use of Laboratory Animals and were authorized by the Ethics Committee of the First Affiliated Hospital of Zhengzhou University and the Ethics Committee of Zhengzhou University. Adult male Sprague–Dawley (SD) rats (200–250 g) were raised in separated cages and provided with water and food. One hundred SD rats were randomly assigned to four groups: Sham, SCI rats treated with PBS (SCI+PBS), SCI rats treated with EV-free CM (SCI+ EV-free CM), and SCI rats treated with BMSC- EV (SCI+BMSC-EV). SCI SD rats were anesthetized with 4% isoflurane and anesthesia was maintained with 2% isoflurane for 2 min (1 L/min). When the rats were unconscious, the skin, fascia, and muscle were bluntly separated to allow a laminectomy to be performed at the level of T10. The exposed spinal cord underwent a contusive injury (200 kilodyne) with a spinal cord impactor (IH Impactor; Precision Systems and Instrumentation, Lexington, KY, United States). After surgery, rats were given bladder care until they could urinate spontaneously. The sham group was subjected to laminectomy without contusive injury.

### BMSC-EV and EV-Free CM Injection

In the SCI+ EV-free CM group, 200 μL EV-free CM derived from 1 × 10^6^ MSCs were injected via the tail vein (200 μL/min) 30 min after SCI. At 1-day post-injury (dpi), 200 μL of EV-free CM was injected in the same manner. In the SCI+BMSC-EV group, 200 μL of EV derived from 1 × 10^6^ BMSCs was injected into the tail vein (200 μL/min) at 30 min post-SCI. Subsequently, 200 μL of EV (200 μg/mL) were injected in the same manner at 1 dpi. In the SCI group, 200 μL PBS was injected at the same timepoints mentioned above.

### TUNEL Assay

At 1 dpi, spinal cord cell apoptosis within the lesion was identified and quantified with an *in situ* cell death detection kit (Roche, Mannheim, Germany) according to the manufacturer’s protocol. First, tissue slides were dewaxed and rehydrated, and this was followed by pre-treatment of the tissue slides with proteinase-K for 30 min. Then, tissue slides were equilibrated with the kit’s equilibration buffer for 1 h at 37°C. After incubation with converter POD, tissue slides were incubated with diaminobenzidine and imaged using a microscope at 200× magnification (Olympus, Tokyo, Japan).

### Nissl Staining

Nissl staining was used to assess neuronal survival. Briefly, the tissue slides were incubated with 1% thionine solution at 50°C for 40 min, and subsequently differentiated with 70% alcohol for 3 min.

### Immunofluorescence

First passage pericytes were grown in culture dishes with a cell creep plate. At 28 dpi the rats were sacrificed and perfused with warm PBS and subsequently with 4% paraformaldehyde in PBS solution. 5% BSA was applied for 1 h to block non-specific antibody binding, followed by 0.3% Triton X-100 in PBS for 30 min. All paraffin sections of tissues and cell creep plates were incubated with primary antibodies at 4°C overnight. These included: rabbit anti-platelet-derived growth factor receptor beta (PDGFR-β; 1:200; Abcam), mouse p65 (1:200; Cell Signaling Technology, Danvers, MA, United States), rabbit anti-Type I collagen (Col-I; 1:200; Abcam), and mouse anti-NF200 (1:100; Abcam). All paraffin sections of tissues and cell creep plates were rewashed with PBS three times and subsequently incubated with their corresponding secondary antibodies, including red cyanine-3-conjugated goat anti-rabbit IgG (1:500; Beyotime Biotechnology, Shanghai, China) and green 488-conjugated goat anti-mouse IgG (1:500; Beyotime Biotechnology) for 2 h at 37°C. The cell nuclei were stained with diamidino-2-phenylindole (DAPI) and imaged using microscopy (Olympus, Tokyo, Japan).

### Basso, Beattie, and Bresnahan (BBB) Locomotor Rating Scale

Basso, Beattie, and Bresnahan scores were detected to assess the locomotor function of the hindlimbs after SCI. According to the BBB open-field 21-point locomotor rating scale (Basso et al., 1995), the assessment was conducted weekly and scores were recorded by two independent observers blinded to the treatment groups.

### Assessment of BSCB Permeability With Evans Blue (EB)

According to a well-established protocol adapted from He et al., EB extravasation leakage was performed to evaluate BSCB integrity (He et al., 2017). Briefly, EB (2% in normal saline, 4 mL/kg; Sigma-Aldrich) was injected into uninjured control animals or SCI rats through the tail vein 1 day post-SCI. Rats were anesthetized 2 h later and extensively perfused with saline until the EB dye no longer flowed out of the right atrium to remove the intravascular EB. After isolation of the spinal cord, the meninges were stripped off and the dye distribution was evaluated via photographs taken using a digital camera. The injured spinal cord tissues were weighed and then homogenized in 50% trichloroacetic solution and centrifuged for 20 min at 20,000 × *g*. The supernatant was collected and mixed with absolute ethanol (1:3). EB extravasation was quantified by measuring fluorescence from the supernatant (620 excitation, 680 emission). Ipsilateral/contralateral ratios were calculated and analyzed.

### Cell Isolation and Culture

According to an established protocol (He et al., 2013; Wan et al., 2018), rat spinal cord microvascular pericytes were isolated from SD rats (3–5 weeks old) with some modifications. In brief, the rat spinal cord was separated and minced in ice-cold PBS, followed by digestion in 10 mL Dulbecco’s Modified Eagle’s Medium (DMEM) containing collagenase type 2 (1 mg/mL; Worthington, Lakewood, NJ, United States) and DNase I (15 g/mL; Sigma) for 1.5 h at 37°C. After adding 10 mL DMEM to the cell suspension and centrifuging it at 500 × *g* for 5 min at 4°C, the sediment was suspended with 20% BSA-DMEM and centrifuged (1000 × *g*, 20 min) to adsorb the neurons and myelin. The sediment containing microvessels were digested with collagenase/dispase (1 mg/mL; Roche, Basel, Switzerland) and DNase I (6.7 mg/mL) in DMEM for 1 h at 37°C. A 33% continuous Percoll (GE Healthcare, Buckinghamshire, United Kingdom) gradient was used to discard endothelial cells and purify pericytes. After centrifugation at 1000 × *g* for 10 min, the pellet was collected for plating in culture dishes coated with collagen type IV (both 0.1 mg/mL; Sigma-Aldrich). Pure rat spinal cord pericytes were obtained by a prolonged 2-week culture protocol, during which endothelial cells disappeared. Cells were incubated in suitable conditions (5% CO_2_, 95% O_2_) at 37°C and the culture medium was changed every 3 days. The fourth to sixth passages of purity exceeding 95% were used.

### Oxygen-Glucose-Deprivation/Reperfusion (OGD/R)

Cells were exposed to oxygen-glucose-deprivation/reperfusion (OGD) conditions as per a previously reported protocol with modifications. Briefly, the glucose-free medium was placed in a hypoxia chamber (37°C, 1% O_2_, 5% CO_2_, and 94% N_2_) for 30 min to remove oxygen from the medium. Cells were washed with glucose-free medium twice and then incubated in the prepared hypoxia medium for 2 h in the hypoxia chamber. After 2 h of OGD exposure, cells were reperfused with oxygen and the corresponding medium according to a previous protocol (Wan et al., 2018).

### Migration Assay

A *trans*-well assay was used to assess the migration capacity of pericytes. Briefly, a volume of 200 μL (1 × 10^5^ cells/cm^2^) of pericytes were seeded into the upper chamber with 600 μL/well of 0 or 100 μg/mL of BMSC-EV added to the lower chamber. After OGD/R exposure, cells on the upper surface of the filter membranes were removed gently by a cotton swab, while cells migrating to the lower surface of the filter membrane were fixed with 4% paraformaldehyde for 15 min before staining with 0.1% crystal violet for 30 min. The number of stained cells was counted under an optical microscope (Olympus, Tokyo, Japan).

Pericytes (2 × 10^5^ cells/well) were seeded into 6-well plates for 48 h until the cells had grown to 100% confluence. A uniform scratch was made by sterile 200 ml pipette tip in the well. The eliminated cells were washed away with PBS three times and then BMSC-EV was added. Initial images were acquired microscopically. Images were taken at 0 and 6h after OGD/R exposure. The migration was determined using Image Pro Plus 6.0 (IPP, Media Cybernetics, Carlsbad, CA, United States) as an average closed width of the wound relative to the initial wound width.

### Drug Treatment

Pyrrolidine dithiocarbamate (PDTC; Sigma-Aldrich) was dissolved to 10 μM with dimethyl sulfoxide (DMSO) and added to pericytes 3 h before OGD exposure to inhibit the nuclear factor kappa-light-chain-enhancer of activated B cells (NF-kB) signaling pathway.

### Western Blot Analysis

Total protein was extracted with the RIPA lysis buffer (Beyotime Biotechnology) containing 1% phenylmethane sulfonyl fluoride (PMSF). Proteins from the cytoplasm and nuclear fraction were separately extracted using a nuclear protein extraction kit (R0050; Solarbio). A BCA Protein Assay Kit (23227; Solarbio) was used to quantify protein concentrations. Subsequently, proteins were denatured by boiling, separated with 10% sodium dodecyl sulfate polyacrylamide gel electrophoresis (SDS-PAGE), and transferred onto a polyvinylidene fluoride (PVDF) membrane. This was immersed in non-fat milk powder (5%) for 2 h at room temperature to block non-specific binding sites on the membrane. Next, the PVDF membrane was incubated with primary antibodies against p65 (1:1000; #8242; Cell Signaling Technology), phosphorylated-p65 (1:1000; #3033; Cell Signaling Technology), IKBα (1:1000; #4812; Cell Signaling Technology), p-NF-KB inhibitor alpha (p-IKBα; 1:1000; #2859; Cell Signaling Technology), β-actin (1:1000; 20536-1-AP; Proteintech), and Histone H3 (1:1000; #9715; Cell Signaling Technology) at 4°C overnight, followed by the secondary goat anti-mouse or rabbit HRP–linked antibodies. Target bands were visualized by enhanced chemiluminescence reagent (Thermo Fisher Scientific, United States) and analyzed using Image J.

### Statistical Analysis

Statistical analyses were performed using Graph Pad Prism 5.0 (San Diego, CA, United States) in triplicate. Differences with *P* < 0.05 were considered statistically significant. All data are presented as mean ± standard error of the mean (SEM). BBB scores were analyzed using a two-way analysis of variance with repeated measures. Statistical analysis between two groups was conducted using Student’s *t*-tests and Bonferroni *post hoc* tests.

## Results

### Extracellular Vesicles Attenuated Neuronal Cell Death and Improved Motor Recovery After SCI *in vivo*

**Figure 1:**
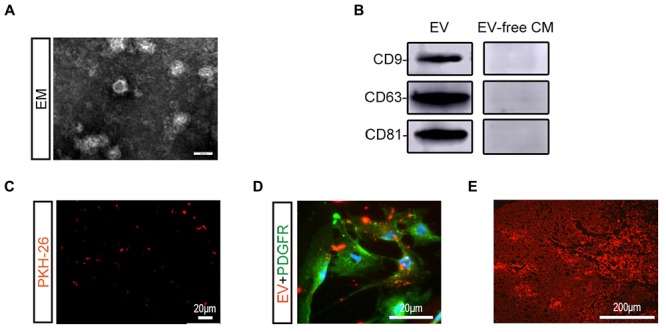
Characterization and labeling of BMSC-EV. **(A)** Morphological observation of BMSC-derived extracellular vesicles (BMSC-EV) by TEM. Scale bar = 100 nm. **(B)** Expression of BMSC-EV markers (CD9, CD63, CD81) was detected using western blot analysis. **(C)** Immunofluorescence image showing the PKH26-stained BMSC-EV (red). Scale bar = 20 μm. **(D)** Immunofluorescence image showing the PKH26-stained BMSC-EV (red) were taken up by pericytes (green). Scale bar = 20 μm. **(E)** Immunofluorescence image showing PKH26-labeled BMSC-EV (red) reaching the SCI lesion. Scale bar = 200 μm.

BMSC-EV were isolated in BMSC-culture supernatant as previously described and further identified by TEM and western blot analysis (Figure 1A,B). Under the TEM, BMSC-EV had a round-shaped morphology. Unique exosome markers, including CD63, CD9, and CD81 were also confirmed by western blot analysis, while exosome-specific proteins were not detected in EV-free CM. To trace BMSC-EV to their target tissue, BMSC-EV were labeled in red with PKH-26 before injection (Figure 1C). As shown in Figure 1D,E, numerous BMSC-EV were taken up by pericytes and reached the spinal cord lesion.

**Figure 2:**
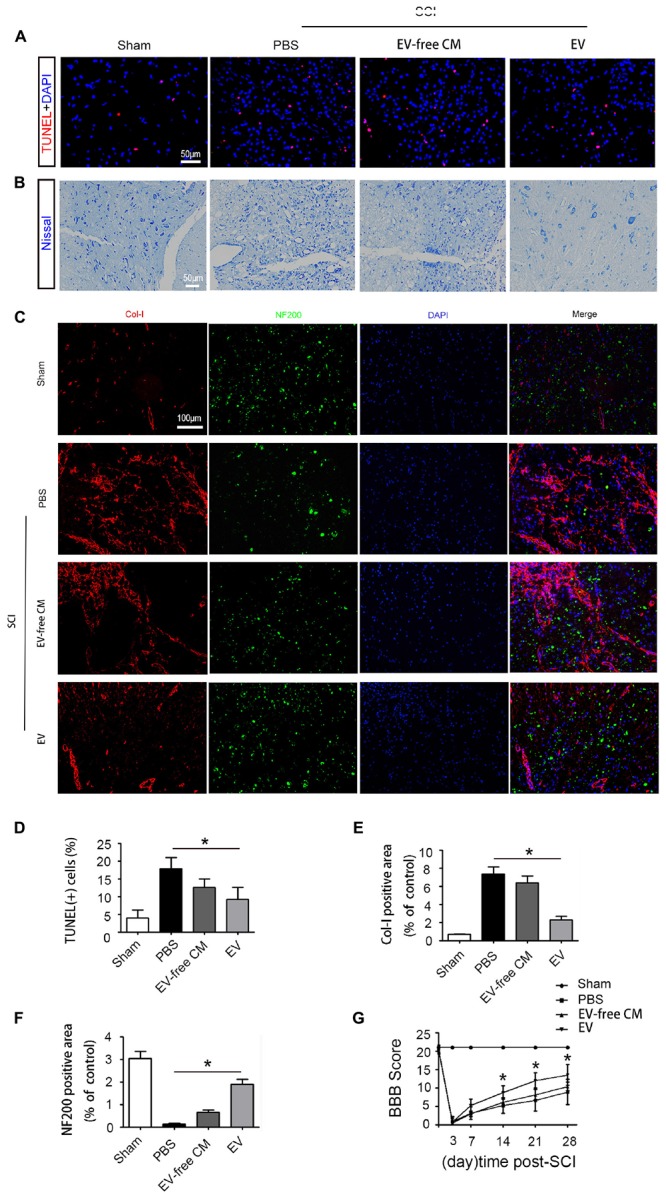
Exosomes improve cell morphology and motor function recovery after SCI *in vivo*. **(A)** TUNEL assay showing fluorescence images of TUNEL-positive apoptotic cells (red) after SCI treated with EV-free CM or BMSC-EV. Cell nuclei were stained with DAPI. Scale bar = 50 μm. **(B)** Nissl staining showing the transverse sections of the spinal cord at 28 dpi (*n* = 6). Scale bar = 50 μm. **(C)** Immunofluorescence staining for Col-I (red) and NF200 (green) in the transverse sections at 28 dpi from different groups. Scale bar = 100 μm. **(D)** Comparison of the number of TUNEL-positive cells with EV-free CM or BMSC-EV treatment (*n* = 6). ^∗^*P* < 0.05. **(E,F)** Semi-quantitative analysis of the percentage of Col-I (red) and NF200 (green) positive areas in each group (*n* = 6). ^∗^*P* < 0.05. **(G)** Representative Basso, Beattie, and Bresnahan (BBB) scores of rats from different groups at different timepoints (*n* = 10). ^∗^*P* < 0.05.

To explore the role of BMSC-EV, we performed a series of histology analyses. First, TUNEL assays were used to assess neuronal cell death in the traumatic area of the spinal cord *in vivo*. At 1 dpi, a dramatic reduction in the number of TUNEL-positive cells was found in the BMSC-EV group compared with the SCI+PBS group and EV-free CM treated group (Figure 2A,D). Next, we assessed the survival of motor neurons in the lesion core using Nissl staining. After BMSC-EV treatment, the number of Nissl-positive neurons significantly increased. This is suggestive of functional recovery (Figure 2B). In summary, these data indicated that BMSC-EV exert an anti-cell death effect.

In addition to glial scars around the lesion, stromal scars at the core of the lesion can also obstruct axon regeneration. Therefore, we further explored the density of neurons and axons in the injured lesion core. Neurofilaments are proteins that specifically exist in neuronal cell bodies and axons and play an important role in maintaining neuronal function and axoplasmic transport. Col-I is produced by pericytes in the core of the lesion, hindering neuronal regeneration. Immunostaining analysis of the 200 kDa subunit of a neurofilament (NF200) and Col-I was performed to assess the density of neurons and axons. At 28 dpi, the staining intensity of NF200 was dramatically reduced while that of Col-I was increased. Interestingly, BMSC-EV administration increased the immunostaining for NF200 but decreased that for Col-I. However, EV-free CM treatment did not alter this effect, indicating that BMSC-EV improved axonal regeneration (Figure 2C,E,F).

To further investigate the effect of BMSC-EV on motor function, hindlimb locomotor behavior was assessed using the 21-point BBB locomotor rating scale in an open-field arena. Both hindlimbs were totally paralyzed (BBB score = 0) at 3 dpi. However, rats in the BMSC-EV group exhibited significant improvements in locomotor function compared with rats in the SCI+PBS and EV-free CM groups from the second week post-SCI (Figure 2G).

### BMSC-EV Administration Increases BSCB Pericyte Coverage and Decreases BSCB Permeability

**Figure 3:**
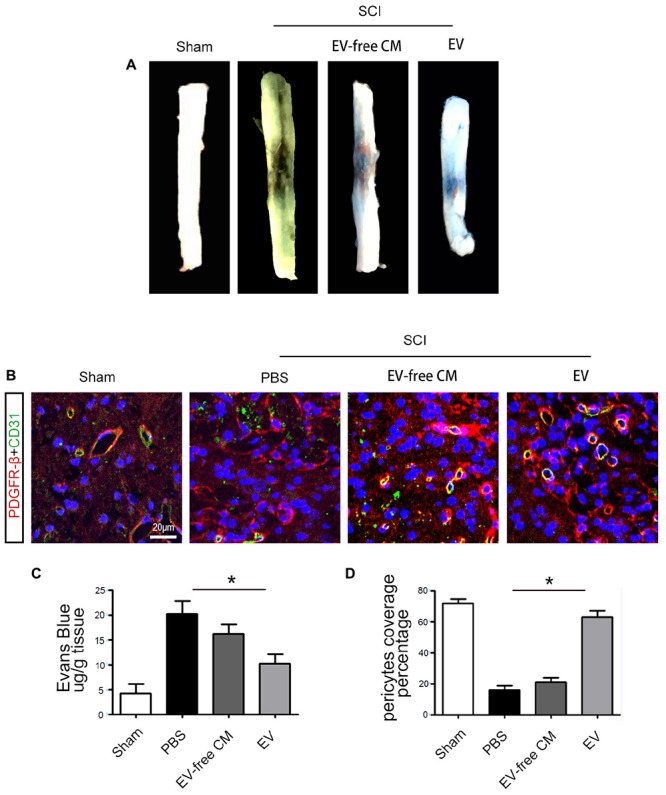
BMSC-EV administration protects the Blood-Spinal Cord Barrier (BSCB). **(A)** Representative gross morphology of Evans Blue (EB) extravasation from different groups. **(B)** Immunolocalization microscopy analysis of CD31+ endothelial cells and PBGFRβ+ pericytes after SCI and treatment with BMSC-EV. Scale bar = 20 μm. **(C)** The effects of BMSC-EV on BSCB permeability represented by quantification of extravascular EB after SCI (*n* = 6). ^∗^*P* < 0.05. **(D)** Quantification of regional PDGFRβ-positive pericytes coverage of endothelial cells in spinal cord capillaries (*n* = 6). ^∗^*P* < 0.05.

Extracellular vesicles promoted functional recovery by directly acting on neurons, in addition to other effects. The microvasculature and the BSCB were damaged immediately after SCI, peaking in the first week and persisting for up to 6 weeks. Therefore, we measured EB infiltration to determine BSCB permeability. The results proved that the amount of EB in the surrounding tissue was significantly increased after SCI compared to the sham group, and that BMSC-EV reduced the EB extravasation, indicating preservation of BSCB integrity (Figure 3A,C).

To further investigate the mechanisms underlying the protection of BSCB integrity by BMSC-EV, we examined pericytes. Pericytes occupy a strategic position in the neurovascular unit, crucially contributing to the structure and function of the vascular bed. Previous studies showed that 5 days after SCI, some pericytes had detached from the vascular wall. Thus, we quantified the percentage of vessels with PDGFRβ-positive pericyte coverage to assess pericyte/vessel contact 5 days after SCI. First, the morphology of PDGFRβ-positive cells after SCI differed from pericytes in the uninjured sham control group. Consistent with previous studies, the vascular wall showed a loss of pericyte coverage, and the morphology of the pericytes differed from those in the uninjured sham controls. Interestingly, our findings demonstrated greater preservation of vascular pericyte coverage after BMSC-exo administration. In contrast, there was no significant effect of exo-free CM treatment (Figure 3B,D).

### BMSC-EV Inhibit Pericyte Migration via the NF-κB p65 Pathway

**Figure 4:**
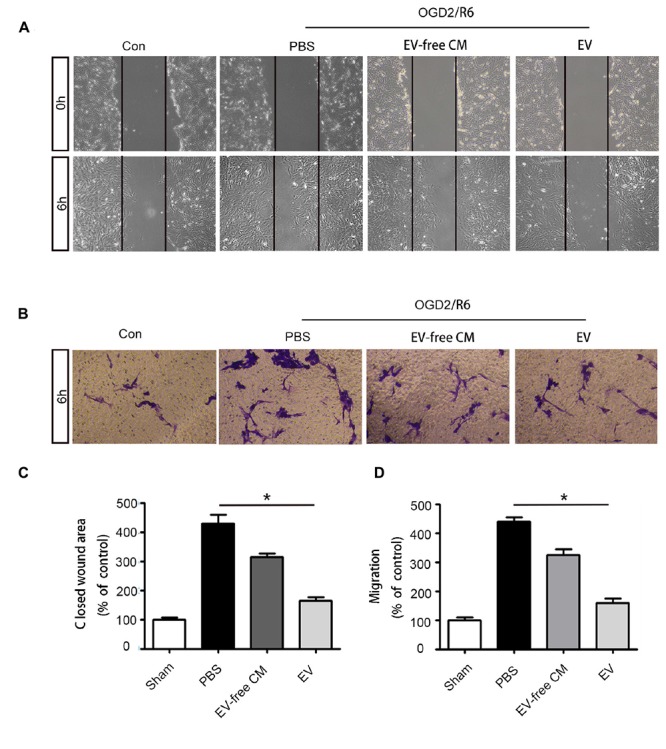
BMSC-EV regulate pericyte migration. **(A,C)** Pericyte migration was detected by healing assay initial images (0 h) and after OGD/R treatment (original magnification 200×) (*n* = 6). ^∗^*P* < 0.05. **(B,D)** Pericyte migration was detected by a Transwell migration assay after (Oxygen-glucose-deprivation/reperfusion) OGD/R treatment (original magnification 100×) (*n* = 6). ^∗^*P* < 0.05.

Based on the *in vivo* results, we further explored the effect of BMSC-EV on pericytes *in vitro*. Many studies have shown that abnormal migration of pericytes results in an increase in BBB permeability (Pfister et al., 2008; Wan et al., 2018). Thus, we used OGD/R to simulate the pathological environment of SCI *in vitro*. Our data revealed that pericyte migration was enhanced after OGD/R exposure. This migration could be partly attenuated by BMSC-EV administration, as assessed by a wound healing assay and transwell migration assay (Figure 4).

**Figure 5:**
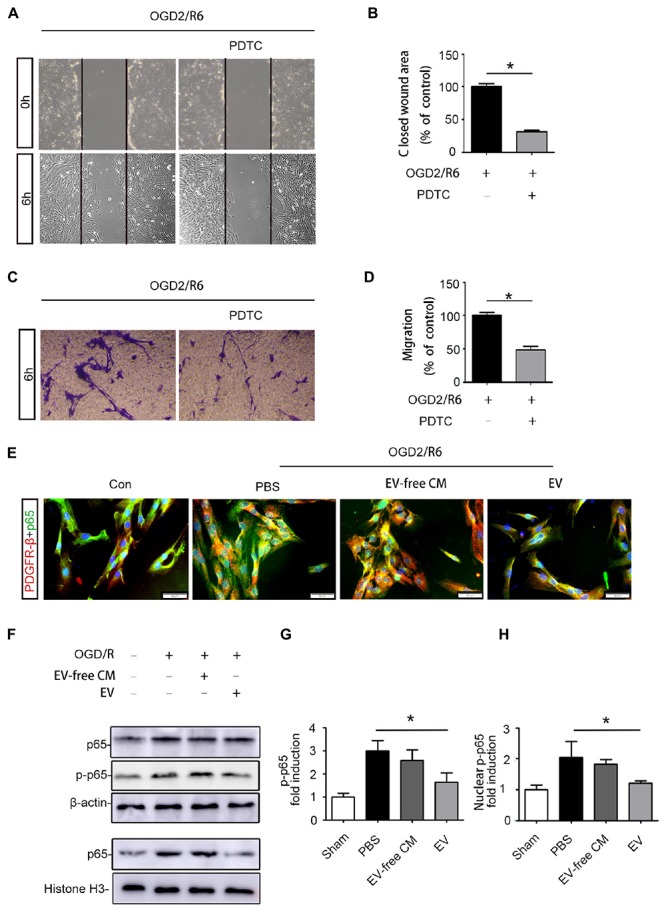
BMSC-EV regulate pericyte migration via NF-kB p65 signaling. **(A,B)** Pericyte migration was prohibited by PDTC under the OGD/R condition, as determined by the wound healing assay (original magnification 200×) (*n* = 6). ^∗^*P* < 0.05. **(C,D)** Pericyte migration was prohibited by PDTC under the OGD/R condition, as detected by a Transwell migration assay (original magnification 100×) (*n* = 6). ^∗^*P* < 0.05. **(E)** Immunofluorescence images showing the proportion of p65 entering the nuclei of pericytes in different groups (original magnification 400×). ^∗^*P* < 0.05. **(F,G,H)** Western blot analysis of the total protein expression of p-NF-kB p65 and NF-kB p65 in pericytes from different groups (*n* = 6). ^∗^*P* < 0.05.

**Figure 6:**
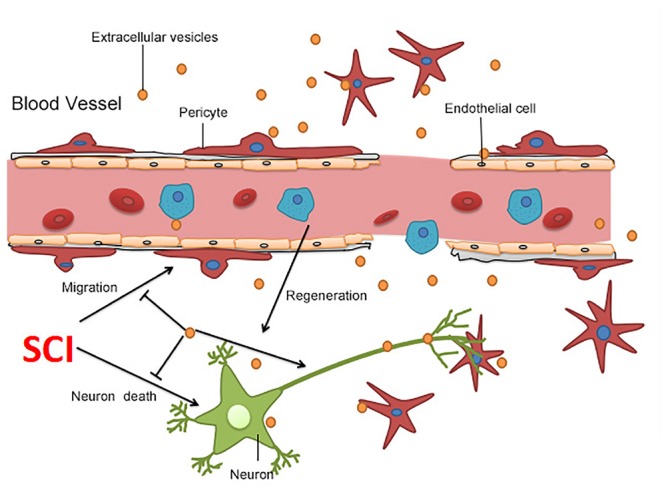
Summary of the proposed mechanism of BMSC-EV-induced protection after SCI. BMSC-EV have the capacity to repress neuronal cell death, promote neuronal survival, improve axonal regeneration, and therefore boost locomotor recovery after SCI. Moreover, BMSC-EV protected the BSCB through suppression of pericytes detaching from the microvascular wall.

We further explored the molecular mechanisms underlying BMSC-EV-mediated migration of pericytes. Numerous studies have confirmed that the NF-kB signaling pathway regulates the migration of various cancer cells (Shostak and Chariot, 2015). Thus, we hypothesized that BMSC-EV regulate pericytes migration through NF-kB signaling. To confirm that the NF-kB signaling pathway participates in the migration of pericytes, we pre-treated pericytes with PDTC, an NF-kB inhibitor before subjecting them to OGD/R. PDTC dramatically decreased the activation of the NF-kB signaling pathway in pericytes as well as the migration of pericytes after OGD/R exposure, as assessed by the Transwell migration and wound healing assay (Figure 5A–D). Our results demonstrated that the total protein expression of p-p65 and p65 were enhanced after OGD/R. At the same time, the expression of the nuclear protein NF-kB p65 was increased in pericytes. Compared with EV-free CM treatment, BMSC-EV caused greater inhibition of this increase in pericytes (Figure 5F–H). Similarly, immunofluorescence staining revealed the rate at which p65 entered the nucleus was decreased via BMSC-EV treatment (Figure 5E). This indicated that pericyte migration was enhanced through activation of the NF-kB signaling pathway and that BMSC-EV inhibited pericytes migration after OGD/R treatment, while EV-free CM did not have this beneficial effect. Collectively, our data suggest that BMSC-EV improve the integrity of BSCB and protect neuronal cells (Figure 6).

## Discussion

Spinal cord injury can result in paralysis, bladder dysfunction, and even death. However, functional recovery is impeded by neuronal necrosis, ongoing neuroinflammation, the altered microenvironment, and a dysfunctional BSCB (Kjell and Olson, 2016). This highlights the need for effective treatments. Exosomes are essential components of the paracrine factors released by MSCs (Ren, 2018). Our findings demonstrate that BMSC-EV administration critically improves neuronal regeneration and attenuates nerve cell death, which is beneficial for motor function recovery after SCI. Furthermore, pericyte migration could be prohibited by BMSC-EV administration via suppression of the activation of the NF-kB signaling pathway. This led to an improvement in the integrity of the BSCB.

Due to their potential capacity to self-reproduce and undergo multidirectional differentiation, alongside their low immunogenicity and regenerative secretion profile, MSCs have attracted a great deal of attention. Furthermore, cell transplantation treatments have been applied in clinical trials of Parkinson’s disease (Cizkova et al., 2011; Venkatesh and Sen, 2017). In addition, MSC therapy in animal models of SCI promoted recovery of motor function (Shende and Subedi, 2017). However, in this study a large number of transplanted BMSCs got trapped in the lung or liver, meaning that few of the MSCs were transferred to the target lesion (Phinney and Prockop, 2007). Thus, there are several limitations and difficulties that need to be addressed before wide clinical application of MSCs. However, recent studies have shown that one potential mechanism driving the beneficial effects of MSCs administration may be the secretion of a large spectrum of molecules rather than the transplanted-MSCs directly differentiating into desired cells (Ratajczak et al., 2014).

EV are one of the most important components of this paracrine secretion. MSC-derived EV contain several important active components, and their double lipid membrane also protects the inner contents from degradation and digestion (Marote et al., 2016; Yang et al., 2017). Furthermore, EV can freely pass through the BSCB, which facilitates their ability to reach the lesion. All of these are beneficial for functional recovery after SCI (Liu et al., 2018). Therefore, we chose BMSC-EV for SCI in this study. We successfully isolated EV from BMSCs, which was confirmed by TEM and labeling with specific exosome surface markers, including CD9, CD63, and CD81. Furthermore, our results demonstrate that BMSC-EV administration contributed to neuronal survival, with less apoptosis and promotion of functional recovery observed.

The BSCB plays an essential role in supporting proper nervous system function and is modulated by neurovascular unit cells to maintain its unique properties and function (Bartanusz et al., 2011). Pericytes, part of the neurovascular unit, play a vital role in vascular integrity and barrier properties during development and physiological conditions (Sweeney et al., 2016). Previous studies demonstrated that pericytes participate in maintaining microvascular stability mainly via three potential mechanisms: regulation of vesicular transport and bulk flow transcytosis, promotion of endothelial tight junction protein expression (i.e., ZO-1 and occludin), and moderate tight junctional alignment (Jo et al., 2013). In neurological conditions, such as stroke and amyotrophic lateral sclerosis (ALS), mounting evidence has demonstrated that abnormal migration of pericytes contributes to further enlargement of the lesion (Winkler et al., 2013; Cheng et al., 2018). Blood vessels at the injury site are immediately destroyed after SCI and even the BSCB distant from the injury core was permanently disrupted (Figley et al., 2014). SCI causes pericytes to detach from the vascular wall, leading to microvascular stability disruption and an increase in BSCB permeability. Here, our findings demonstrate that extracellular vesicles from BMSCs improve the structural integrity of the BSCB by inhibiting the migration of pericytes and improving the rate of pericyte coverage. As a result, better functional recovery after SCI was obtained by BMSC-EV treatment.

In the resting state, NF-κB signaling participates in the regulation of proliferation, differentiation, migration, survival, and other key cellular activities. Multitudinous pro-inflammatory agents induced by SCI, such as cytokines, free radicals, and reactive oxygen species, can activate NF-κB, which leads to an inflammatory response and neurotoxic effects by glial cells (Rahimifard et al., 2017). Neuroinflammation and ischemia induce two different types of reactive astrocytes, namely “A1” and “A2” (analogy to the “M1”/“M2” macrophage nomenclature), and A1 astrocytes might be harmful to synapses (Liddelow et al., 2017). Previous studies demonstrated that A1 astrocytes that are activated via NF-κB signaling impede functional recovery after SCI (Wang et al., 2018). Furthermore, activation of NF-κB in BV2 microglia leads to the production of multiple pro-inflammatory cytokines (Blasi et al., 1990; Zhou et al., 2016). In our study, NF-kB p65 in pericytes was increasingly phosphorylated and the amount of NF-kB p65 within the nucleus was elevated after exposure to OGD/R. At the same time, OGD/R promoted pericyte migration, while PDTC inhibited it. Thus, all of this demonstrates that the pericytes migrate away from the vascular wall via activation of NF-κB signaling. In addition, we have shown for the first time that exosome administration could significantly suppress abnormal migration of pericytes via inhibition of the NF-kB signaling pathway. This suggests a potential mechanism whereby pericyte coverage is enhanced at an early stage of SCI, as assessed via the number of pericytes adhering to endothelial cells.

Overall, our findings are in accordance with results reported by previous studies, which have revealed that MSC-derived extracellular vesicles promote neurovascular remodeling as well as reducing neuroinflammation after SCI or traumatic brain injury (Kim et al., 2016; Zhang et al., 2017; Liu et al., 2018). Furthermore, Li and colleagues found that exosomes derived from microRNA-133b-modified MSCs improved the survival of neurons and the regeneration of axons via extracellular signal-regulated kinase 1/2 (ERK1/2), signal transducer and activator of transcription 3 (STAT3), and cAMP response element binding protein (CREB) signaling pathways (Li et al., 2018). The extracellular vesicles components involved in the regulation of pericyte migration remain to be elucidated. Moreover, we only detected the migration of pericytes 6 h after OGD exposure. Such a time period makes it impossible to identify the exact effect of BMSC-EV. Thus, observations at longer timepoints should be performed in future experiments.

In summary, our study provides the first evidence that BMSC-EV can effectively inhibit the migration of pericytes, thereby maintaining the integrity of the BSCB after SCI. This was accompanied by a reduction in neuronal cell apoptosis, axonal regeneration, and locomotor recovery. Therefore, our results suggest that extracellular vesicles may serve as a promising treatment strategy for SCI.

## Data Availability

All datasets generated for this study are included in the manuscript and/or the supplementary files.

## Author Contributions

YJ conceived and supervised the research. YLu and YZ designed and planned the experiments. YLu, YZ, LW, and KW performed the experiments. YLi, RD, and YY were involved in collecting the data and statistical analyses. YLu drafted the manuscript and figures. YZ, RZ, and KW reviewed the manuscript.

## Conflict of Interest Statement

The authors declare that the research was conducted in the absence of any commercial or financial relationships that could be construed as a potential conflict of interest.
